# S124. RECOVERY TRAJECTORIES IN FIRST EPISODE PSYCHOSIS PATIENTS: THE ROLE OF TIMING

**DOI:** 10.1093/schbul/sby018.911

**Published:** 2018-04-01

**Authors:** Rosa Ayesa-Arriola, Jose Maria Pelayo-Teran, Esther Setien-Suero, Rocio Perez-Iglesias, Benedicto Crespo-Facorro

**Affiliations:** 1 Marqués de Valdecilla University Hospital, IDIVAL, School of Medicine, University of Cantabria; 2 Servicio de Psiquiatría. Hospital El Bierzo SACYL Ponferrada, Castilla y León; 3 University Hospital Marques de Valdecilla -IDIVAL, University of Cantabria, CIBERSAM; 4 IoPPN, King’s College London

## Abstract

**Background:**

Prevent symptom relapse and promote functional recovery are the two main goals of early intervention services in first episode of psychosis. Identify patterns of recovery will be important in developing and implementing targeted recovery focused interventions. The goal of this study was to explore trajectories of recovery following a first episode of psychosis.

**Methods:**

A sample of 373 FEP patients was followed over 3 years. Recovery profiles in terms of symptomatic and functional remission were explored. Relapses during follow-up were considered.

**Results:**

Four recovery trajectories were identified: good stable (26%), good unstable (21%), poor unstable (10%), poor stable (43%). Those who met criteria for good stable recovery more likely have less severe baseline negative symptoms (OR= 2.092; 95% CI = 0.99–4.419) and not be diagnosed with schizophrenia (OR= 2.242; 95% CI = 1.015–4.954); short DUP (OR= 2.152; 95% CI = 0.879–5.27) and low premorbid IQ (OR = 2.281; 95% CI = 0.954–5.457) increased the likelihood of good unstable recovery; less severe baseline negative symptoms (OR= 3.851; 95% CI = 1.422–10.435) and single status (OR= 4.307; 95% CI = 1.014–18.293) increased the likelihood of a poor unstable recovery when these three trajectories were compared with a poor stable recovery. Poor unstable trajectory was significantly associated with a high relapse rate (73%).

**Discussion:**

Our results shed light on identifying different recovery profiles in FEP. Despite evidence for early intervention effectiveness, we should explore ways to prevent relapse and improve long-term recovery, particularly attending the role of timing in the design of interventions.

